# Queervertising: An empowerment tool for the gay men and lesbian community

**DOI:** 10.12688/f1000research.126882.1

**Published:** 2023-03-17

**Authors:** Patricia P. Iglesias-Sánchez, Carmen Jambrino-Maldonado, Carlos de las Heras-Pedrosa, Rafael Díaz-Tendero

**Affiliations:** 1 Department of Business Administration, Faculty of Commerce and Management, University of Malaga, Malaga, 29071, Spain; 2 Department of Audiovisual Communication and Advertising, University of Malaga, Malaga, 29071, Spain

**Keywords:** Queer, Advertising, Gay men and lesbians’ community, LGBTIQ+, Brands, gender identity, GLBTQ, LGBT

## Abstract

**Background:** Today’s society clams to be more inclusive, but there has been a lack of practical examination of this area. This study analyses how advertising and society interact and evolve in parallel, with advertising seeking to balance more traditional representations – in accordance with the Mirror Theory – with mainstreaming, which can influence social change. In this case, analysis is focused on the homosexual community.

**Methods:** A content analysis of audiovisual advertising in Spain from the 1960s to 2021 is carried out in addition to a review of historical milestones and legislation.

**Results:** The results evidence the transformation of advertising. The main findings show a shift from the total invisibility of the gay men and lesbian community in the 1960s to effective and respectful integration today.

**Conclusions**: Queervertising is proposed as a new theoretical concept as the result of gender and sexual diversity being identified in advertising over time. The inclusion of gay men and lesbians in advertising is a current trend that, moreover, offers a challenge for brands. Although this turnaround in advertising creativity should be highlighted and recognized as being to some extent responsible for changes and social evolution, the commercial messages which are found today are still not always disruptive or excessively explicit, in order to avoid some rejection by audiences.

## Introduction

Minorities have captured the attention of researchers from different disciplines although the societal impact from their representations in advertising remains a current question under exploration ( Branchik & O’Leary, 2016; McDonald *et al.*, 2020; Taylor & Costello, 2017; Yu, 2018; Zmuda, 2014). The dynamic interaction between society and advertising was pointed out by Pollay (1986, p. 18): “Advertising is without a doubt a formative influence in our culture”, and continues to be supported by recent research, such as by Akestam *et al.* (2017) or McDonald *et al.* (2020), who assert that advertising reflects the times as well as social norms. In the same way that values change, advertising adapts and connects with the society on which it depends ( Shinoda *et al.*, 2021). In particular, the treatment of women in advertising and the representation of stereotypes and roles, as well as women’s evolution and ability to contribute to their empowerment, has been extensively explored ( Grau & Zotos, 2016), even introducing the concept of femvertising by Bahadur (2014). By extension, racial minorities have been addressed ( Zmuda, 2014) and more recently the inclusion of gay men and lesbians in advertising imagery ( Hetsroni, 2011; Holz Ivory, 2019; Iribure Rodrigues & Gallina Zanin, 2014; Madinga *et al.*, 2020; McDonald *et al.*, 2020; Oakenfull *et al.*, 2008).

It is precisely the gay men and lesbian community that inspires the development of this research work. This group has also traditionally been stigmatized or made invisible in advertisements ( McDonald *et al.*, 2020; Ragusa, 2005). The purpose of this research is to delve deeper into how advertising and society interact with respect to the inclusion and positive treatment of gay men and lesbians, along with exploring the development of the group itself and the progressive recognition of their rights.

To achieve this objective, a content analysis of audiovisual advertising from 1960 to 2021 in Spain is used. Not only is representation quantified, but also, images and messages regarding sexual and amorous diversity are analyzed.

This study suggests that advertising simultaneously reflects the society of the moment while contributing to its evolution by promoting relevant social changes: tolerance, social inclusion, equality, women´s empowerment. In this case, the focus is normalization and inclusion of the LGBT community. Beyond advertising that has a cause, it is assumed that advertisers’ interest in connecting with the gay men and lesbian community and the ethics of the brands themselves results in the use of a broader and more inclusive imagery. Consequently, this ‘Queervertising’ that takes place gives rise to a new theoretical concept, to analyse the phenomenon, and it provides a new insight into the core of advertising and social changes for researchers. This means reframing the research of advertising and including gay men and lesbians and contributing with practical ideas about the open-mindedness of brands as they seek to engage with stakeholders. There are identifiable themes of firstly, connecting with the gay and lesbian community and secondly, aligning with the increasingly gay-friendly attitude of society. Overall, Queervertising as a concept extends complexity and is complementary to the implementation of two seemingly opposing approaches by brands. On the one hand, their approach involves reaching out and empathizing with a traditionally marginalized group (LGBTIQ+s) and, on the other hand, searching for a balance because if the group portrayal in ads contradicts some consumer beliefs, the experience can be uncomfortable ( Hetsroni, 2011; Ruggs *et al.*, 2018). This theoretical proposal finds its parallel in femvertising ( Bahadur, 2014) and acknowledges advertising’s ability to empower, support and promote sexual and amorous diversity in a way that is respectful and consistent with the current reality.

This paper is structured as follows. After this introduction, a review of the literature on gay men and lesbian imagery in advertising is conducted. The section then explains the methods of this study. The main results are presented next, followed by a discussion. The conclusions of the work then follow. Finally, the main limitations of the study, its practical implications, and future lines of research are considered.

## Theoretical framework

A review of the literature shows that the most prolific time-frame with regard to the topic of gay men and lesbians and advertising has occurred in the last 10 years, coinciding with greater acceptance and the progressive disappearance of associated stigmas ( Branchik & O’Leary, 2016). From 2000 onwards, the visibility of gay men and lesbians in advertising has been increasing ( Branchik & O’Leary, 2016; Iribure Rodrigues & Gallina Zanin, 2014; McDonald *et al.*, 2020). From this prism, a dual directionality and interaction of society and advertising is assumed, whereby advertising preserves traditional and majority values, as well as becoming a mainstreaming tool that favours social advances ( Åkestam *et al.*, 2017; McDonald *et al.*, 2020; Mikkonen, 2010). In this framework, it is necessary to introduce the premises of the Mirror Theory ( Abrams, 1953; Holbrook, 1987), which defends the idea that advertising reproduces the same patterns, behaviours and customs of the society in which it operates. Likewise, advertising can exercise its social function by bringing about social evolution and changes in favour of special collectives ( Bahadur, 2014; Feng & Wu, 2009; Fowler & Thomas, 2015; McDonald *et al.*, 2020; Zmuda, 2014). Regarding the focus of this research work, according to Schmidt and Stocker (2013, p. 180): “advertising and films evoke the masculine and feminine universe – simultaneously they introduce changes according to the changes experienced in society”. Therefore, advertising represents both the most deeply rooted social standards and those new trends or more progressive scenarios, even favouring their advancement.

### Engagement with the target through the cause

For brands, the positive effect of advertising is undisputed and it is often used to strengthen a connection with the target demographic. Åkestam *et al.* (2017) empirically demonstrate that those ad campaigns that include respectful representation of the LGBTIQ+ community increase the empathy of the recipients towards the company and the rest of society. Beyond the reputational benefit, Cunningham and Melton (2014) conclude that incorporating this theme in the creative axes results in improved business performance. Taylor and Costello (2017) indicate that in addition to improving performance, it contributes to the social responsibility of the brand. However, this effect does not occur as a generalizable maxim for all companies and for all targets, as using a cause for advertising and the consequent linked imagery must be managed with caution, because it can also create a critical environment if controversial or disruptive issues are involved ( Gong, 2020; Holz Ivory, 2019).

### Consumers’ self-concepts and brands

The Social Identity Theory proposed by Taylor and Costello (1985) has been widely used in research works to show that the recognition of a portrayal that involves membership of a social group enhances people’s self-image. Consequently, consumers can use brands to form their self-concept ( Akermanidis & Venter, 2014). Several works evidence the contribution that can be made by advertising imagery to individuals’ sexual identity ( Chae *et al.*, 2016; Gong, 2020; Holz Ivory, 2019; Mikkonen, 2010; Oakenfull *et al.*, 2008). As result of this, the use of gay men and lesbians’ subculture symbols in advertising, that reflect gay/lesbian identity, for instance referencing clothes, language, lifestyle etc, have meaning for the community and generate empathy with them ( Oakenfull *et al.*, 2008). By contrast, controversy can occur. On the one hand, a brand’s image can be potentially harmed when a portion of the customer base is not represented or is negatively represented in ads ( Gong, 2020). On the other hand, the portrayal of gay men and lesbianism in advertising imagery can annoy or inconvenience consumers, precisely because they don’t see themselves as being represented, nor their beliefs, lifestyles etc ( Akermanidis & Venter, 2014; Holz Ivory, 2019; Schmidt & Stocker, 2013). This dilemma for brands is often solved by mixing sexual advertisements that explicitly feature both gay men and lesbians and heterosexual models. Gong (2020, p. 916) points out that having “the portrayal of both models within the same message ensures their effectiveness due to the engagement with particular circumstances, [and the] lifestyle and feelings of each group”. In the same vein, Bond and Farrell (2020) underscore that messages which are not explicit but are likely to be associated with both heterosexual or gay men and lesbians were generally still appealing for all targets.

According to this principle, each person finds a relationship between their self-concept and the environment or situations with which they identify and, by extension, sexual orientation also plays a key role in this respect. However, there is limited empirical research about how gay-themed advertising affects heterosexual consumers’ perceptions ( Holz Ivory, 2019; Madinga *et al.*, 2020). These research works indicate that society has been increasingly accepting of gay men and lesbians’ portrayals in advertising but there are significant peculiarities. For example, Holz Ivory (2019) in her study evidences that heterosexual men showed more negative attitudes towards gay male representation than towards heterosexual and lesbian ads. Madinga *et al.* (2020), for their part, add that the negative perception is higher in the case of heterosexual men than for women. In addition to sexuality, other variables inherent to viewers are analysed. Research works have highlighted that a positive or negative attitude to gay men and lesbians’ portrayal in advertising can be explained due to age (there is more acceptance among young people) ( Akermanidis & Venter, 2014; Schmidt & Stocker, 2013), the country of origin (developing countries are more receptive) ( Akermanidis & Venter, 2014; Feng & Wu, 2009; Madinga *et al.*, 2020), a country having strong traditional religious beliefs ( Hetsroni, 2011), or the existence of gay-friendly attitudes ( Madinga *et al.*, 2020; Mikkonen, 2010; Orr *et al.*, 2005).

### Commercial and economic power of the gay men and lesbians community

The last reason explaining the recent concern for depicting the LGBTIQ+ community in advertising has to do with its interest to companies. On the one hand, there is so-called gay power due to the community’s purchasing power and its being an important part of the population. On the other hand, showing sympathy towards the gay movement attracts gay-friendly groups, which brands also wish to connect to.

It is estimated that 6% of the world's population is LGBTIQ+ ( Jacob, 2017). Specifically, Spain has the second-highest proportion of this group in the world (6.9%), behind only Germany. In addition, Spain holds first place as a gay-friendly country when it comes to its acceptance and defence of gay rights ( Poushter & Kent, 2020). Although there are no official statistics, studies such as that of Oakenfull and Greenlee (2005) show that the gay and lesbian community represents the highest per capita purchasing power of all minority groups in the United States; they even refer to this target as the “pink dollar”. This is a market often idealized by specialists because of its profitability for companies ( Akermanidis & Venter, 2014). Thus, the representation of gay men and lesbians in advertisements aims to connect with this public ( Iribure Rodrigues, 2017; Read *et al.*, 2019) while favouring a brand image that respects gay-friendly targets, who develop a superior feeling for firms that favour the acceptance of the movement in their creative strategy ( Åkestam *et al.*, 2017; Orr *et al.*, 2005).

The challenge for advertisers in Spain, given the high representation of the collective, as well as the country’s categorization as gay-friendly, leads us to assume that in its advertising imagery, the collective’s representation may be significant, and interesting for deeper analysis.

Most studies, in addition to being recent, focus on the effect of advertising content reflecting gay men and lesbians and its consequences for brands: their acceptance/rejection ( Angelini & Bradley, 2010; Branchik & O’Leary, 2016; Descubes *et al.*, 2018; Holz Ivory, 2019; Iribure Rodrigues, 2017; Madinga *et al.*, 2020; Ruggs *et al.*, 2018; Um, 2014), advertising recall ( Angelini & Bradley, 2010; Orr *et al.*, 2005) or even the impact on people’s purchasing intention ( Akermanidis & Venter, 2014; Bond & Farrell, 2020; Holz Ivory, 2019; Orr *et al.*, 2005; Um, 2014). In summary, most of the studies are conducted from a sociological and social perspective, but those that, like this one, focus on the power of advertising to promote a more inclusive society are scarcer ( Gong, 2020; McDonald *et al.*, 2020; Oakenfull & Greenlee, 2005; Ragusa, 2005). It is precisely this gap in the literature that underpins the research questions of this paper:

RQ1. How has advertising imagery evolved with respect to gay men and lesbians since the 1960s in Spain?

RQ2. To what extent do the representations of relationship models that appear accompany the most relevant milestones and legislation in this field?

RQ3. What stance does advertising take on the diversity of love and sexuality in Spain?

## Methods

In order to analyze the interaction between advertising and the advances and social reality of the gay men and lesbian collective, a qualitative approach is chosen, specifically a content analysis of audiovisual advertising spots from 1960 to 2021, in tandem with a review of the most relevant milestones and legislation of the LGBTIQ+ movement in Spain. According to Krippendorff (2004), its use is appropriate for the observation of trends; that is, with this type of analysis we will be able to highlight the changes in advertising over the last six decades.

### Data collection

As mentioned, Spain has the second-highest proportion of LGBTIQ+ citizens in the world and holds first place as a gay-friendly country for its acceptance and defence of gay rights. These reasons lead us to assume that in advertising imagery, the representation of this collective may be significant and interesting for deeper analysis.

The selection of advertising spots was carried out after viewing all the spots collected in the main database of the Spanish Association of Advertisers ( *Asociación Española de Anunciantes*, AEA); in addition, two complementary databases were added to complete the last decade, that of the *El Publicista* journal ( El Publicista, 2021) and the Sanz'Channel ( Sanz, 2016) (N=1408 spots). Finally, our sample consists of 63 spots. Only spots whose message or imagery depicted people and actions representing social relations or events that implicitly or explicitly included sexual and amorous diversity were included. The research has thus tried to identify ads with all the options or relationships beyond heterosexual “conventionality”. It is, overall, a census sample, and consists entirely of commercial communication by brands that directly or indirectly targeted a topic. The sample comprises 63 advertisements because, during our review, this was the total number of relevant audiovisual ads from the total found for the period 1960-2021.

Additionally, it should be added that social campaigns launched by official organizations or associations in defence of LGBTIQ+ rights were excluded, since the object of the research was purely commercial advertising. There were a wide range of brands and kinds of product involved, utilizing content related to the targeted topic: insurance companies, agrifood and drinks businesses, technology and telecom operators, among others ( Table 2). The time period of analysis begins in 1960, coinciding with the first broadcasts of Televisión Española and the increase in the number of households with television sets. The spots are not distributed proportionally in each of the decades overall, due to the low number of relevant advertisements among the total number of ads broadcast over the targeted period. Thus, curiously, the same number of relevant advertisements (5) were identified for each of the decades between 1960-2010. On the other hand, for the period 2010-2021, 38 advertisements reflecting sexual and amorous diversity were found. Two issues should be noted: (1) the underrepresentation of gay men and lesbians in advertising is due to the dictatorial regime that existed in Spain until 1975; and (2), not all advertisements use the family and the couple in their creative strategy. A list of all ads selected can be found in Table 2.

### Measurements, instruments and analysis

The research opted for was a qualitative study using a content analysis combined with a historical analysis to explore the possible relationship between the evolution of advertising with respect to the phenomenon of liberalization and inclusion of the LGBTIQ+ community, and the consequent sociocultural and legislative progress.

Basic information about the product offered, company and sector was extracted for each advertisement. General aspects were analyzed (target, slogan, *etc.*) and finally, details about the treatment of gay and lesbian couples in the ads. Table 1 shows the analysis sheet used for classification. Except for some variables about the identification and classification of images or discourse linked to gay men and lesbians, the variables are in relation with those commonly included in works on the treatment of advertising. Typically, this type of research includes information concerning product, sector, claim, target. The work of Molina Rodríguez-Navas *et al.* (2014) stands out because it laid the foundations of the method of analysis of audiovisual fiction and advertising as sources of knowledge of the past and present. Moreover, they underline the necessity to adapt content analysis to the particular focus of adverts under consideration. For its part, the identification of stereotypes in our approach is mainly supported by variables drawn from the work of Fowler and Thomas (2015) and Olivares *et al.* (2020), specifically variables 7 to 12 and 15 to 17. In any event, our research work involved some adaptation because previous literature focusing on the gay men and lesbian community is really scarce. Consequently, some original variables have been introduced, with the inclusion of variables 13, 14 and 18 in our analysis. The field of study cannot be analyzed without exploring some information about types of couple, and the traditionality of family and love models. The final analysis sheet was the result of a thorough review of literature and a discussion by three researchers in the first stage. After this, the fourth researcher did an analysis test with 5 ads, randomly chosen. The overall process allowed us to refine the analysis sheet for the goals of this research work. The detailed information for each variable used has been included in the calculation sheet shown in Table 1. Results for each advert under examination were recorded using Microsoft Excel spreadsheets in order to proceed to content analysis using the software Keycoder and Cloudwords, and to enable the calculation of some details such as percentages.

**Table 1.** Analysis sheet.

N°	Variable	Description	Scale
1	Product		Type of product
2	Brand		Brand
3	Broadcast decade		1. 1960-1969 2. 1970-1979 3. 1980-1989 4. 1990-1999 5. 2000-2009 6. 2010-2021
4	Sector	Sector	1. Food and beverages 2. Supermarkets 3. Energy and recycling 4. Automotive 5. Telephony 6. Health and beauty 7. Banking and insurance 8. Tourism 9. Entertainment 10. Household appliances 11. Real estate and materials 12. Transportation 13. Other
5	Slogan		Slogan transcript
6	Target	Homosexuals are the target?	1. Yes 2. No
7	Explicit messages	If references are made to homosexuality, are they made in a direct manner or through double meanings and ambiguities?	0. No references to homosexuality are made 1. The message is direct and explicit 2. The message is implicit, using ambiguities
8	Intentionality of the implicit message	If there is, what is this ambiguity used for?	0. No ambiguity 1. Humorous sense 2. To cover a larger target audience and encourage selective perception 3. Other
9	Means to channel implicitness	If there is, what medium is used to play with ambiguity?	0. No ambiguity 1. Image 2. Text
10 ^1^	Argumentative stereotypes	The message contains negative stereotypes about the group	0. No reference is made to homosexuality 1. Yes 2. No
11	Visual stereotypes	Representation of collective negative visual stereotypes	0. No reference is made to homosexuality 1. Yes 2. No
12	Sound stereotypes	The way stereotypical characters speak represents clichés	0. No reference is made to homosexuality 1. Yes 2. No
13	Family and love model	The model of family and couple reflected is …	1. Traditional family and couple model 2. Diverse family and couple models
14	Type of couple	Couple	1. Heterosexual couple 2. Male homosexual couple 3. Female homosexual couple
15	Children	Family with children	1. Yes 2. No
16	Couple’s age	Age	1. No reference is made to the concept of a partner 2. 40 years old or younger 3. Older than 40 years old
17	Gender role	Scale 1 to 5	1. Totally differentiated 2. Sufficiently differentiated 3. Neutral 4. Few differences 5. No differences
18	Historical context	Is the ad congruent with the evolution of advertising and that of the homosexual movement?	1. It is congruent 2. It is not congruent; it is detached from its sociocultural context

Source: Own elaboration

^1^ Variables 10-12 have three categories. The difference between the first and the last option is that the first one will occur mainly in advertisements from the first decades, where homosexuality was a taboo subject. In the latter, homosexuality is presented naturally, without stereotyping.

**Table 2.** List of advertisements chosen.

No	Company	Decade	Link to the advertisement	
1	Cocinas Corcho	1960-1969	Vídeo	https://www.youtube.com/watch?v=EtAL4oxqcRE
2	Muñecas Celeste	1960-1969	Vídeo	https://www.youtube.com/watch?v=65l--qcilC4
3	Yogures Frigo	1960-1969	Vídeo	https://www.youtube.com/watch?v=aB-xK8jg7is
4	Brandy Espléndido	1960-1969	Vídeo	https://www.youtube.com/watch?v=CgSeYzs8D8g
5	Calmante vitaminado	1960-1969	Vídeo	https://www.youtube.com/watch?v=mobC8SlLzd0
6	Nesquik	1970-1979	Vídeo	https://www.youtube.com/watch?v=Skm45m0dwj4
7	Agente FBI de Redondo	1970-1979	Vídeo	https://www.youtube.com/watch?v=HQkOG4JA154
8	Coca-Cola	1970-1979	Vídeo	https://www.youtube.com/watch?v=uM9xov02i0I
9	Coñac Soberano	1970-1979	Vídeo	https://www.youtube.com/watch?v=sP5qrfqyVGo
10	Coñac Soberano	1970-1979	Vídeo	https://www.youtube.com/watch?v=lZRyH9qvJCw
11	Café Monky	1980-1989	Vídeo	https://www.youtube.com/watch?v=2_CwoCR4xZc
12	Scotch Brite	1980-1989	Vídeo	https://www.youtube.com/watch?v=r5xYr4411WM
13	Papillas Riera-Marsá	1980-1989	Vídeo	https://www.youtube.com/watch?v=DnKKnsfSKFc
14	Galletas Princesa	1980-1989	Vídeo (to 37:23)	https://www.youtube.com/watch?v=MU6HK9HYi6k&t=2222s
15	Rociar y Lavar, de Johnson	1980-1989	Vídeo	https://www.youtube.com/watch?v=aS1ZX888aZI
16	Starlux	1990-1999	Vídeo	https://www.youtube.com/watch?v=rlIS0KLnu5g
17	Proyector Famoplay	1990-1999	Vídeo	https://www.youtube.com/watch?v=0J_i861RKg4
18	Renault Laguna	1990-1999	Vídeo	https://www.youtube.com/watch?v=OHSDOP_dnpY
19	CD Rumba Total	1990-1999	Vídeo	https://www.youtube.com/watch?v=DVj6fzXb1_Q
20	Revista Woman	1990-1999	Vídeo (to 26:15)	https://www.youtube.com/watch?v=MU6HK9HYi6k&t=1546s
21	CD Musicón de Verano	2000-2009	Vídeo (to 7:23)	https://www.youtube.com/watch?v=VVqXVfytfzE&t=432s
22	Bosch	2000-2009	Vídeo (to 23:51)	https://www.youtube.com/watch?v=VVqXVfytfzE&t=1411s
23	Ikea	2000-2009	Vídeo	https://www.youtube.com/watch?v=fEU7nfBrqHc
24	Heineken	2000-2009	Vídeo	https://www.youtube.com/watch?v=K0ivMyL9XMI
25	Intereconomía	2000-2009	Vídeo (to 0:41)	https://www.youtube.com/watch?v=YVkjDsCDPhw
26	Coca Cola	2010-2021	Vídeo	https://www.youtube.com/watch?v=OCcGAXEdbnQ
27	Jaguar	2010-2021	Vídeo	https://www.youtube.com/watch?v=Z8CF2Efh-aU
28	Asevi	2010-2021	Vídeo	https://www.youtube.com/watch?v=pHTirii46AY
29	Antena 3	2010-2021	Vídeo	https://www.youtube.com/watch?v=50_R9iftbKQ
30	El Corte Inglés	2010-2021	Vídeo	https://www.youtube.com/watch?v=oRRzOW4yr8w
31	ALDI	2010-2021	Vídeo	https://www.youtube.com/watch?v=-F9bj1k5ysk
32	Meetic	2010-2021	Vídeo	https://www.youtube.com/watch?v=YtgrWAJmr_M
33	Multiópticas	2010-2021	Vídeo	https://www.youtube.com/watch?v=06nVwYKFdkc
34	Desigual	2010-2021	Vídeo	https://www.youtube.com/watch?v=peIaHlZN68w
35	VIPS	2010-2021	Vídeo	https://www.youtube.com/watch?v=cFMi_lbPXOA
36	El Corte Inglés	2010-2021	Vídeo	https://www.youtube.com/watch?v=ehfaRdqmdfg
37	Príncipe	2010-2021	Vídeo	https://www.youtube.com/watch?v=K6R__1oWDRc
38	Multiópticas	2010-2021	Vídeo	https://www.youtube.com/watch?v=9exnCRzfqug
39	Desigual	2010-2021	Vídeo	https://www.youtube.com/watch?v=49Jv7cam900
40	Servihabitat	2010-2021	Vídeo	https://www.youtube.com/watch?v=ZguDuovN_C8
41	Vitaldent	2010-2021	Vídeo	https://www.youtube.com/watch?v=wtCN3mYuulg
42	Élite (Netflix)	2010-2021	Vídeo	https://www.youtube.com/watch?v=Ye6-JBmTUvU
43	La Sexta	2010-2021	Vídeo (to 0:16)	https://www.youtube.com/watch?v=iG8E_GF84qc
44	Vodafone España	2010-2021	Vídeo	https://www.youtube.com/watch?v=rdSSBydol4w
45	Leroy Merlín	2010-2021	Vídeo	https://www.youtube.com/watch?v=TcA0fB2hXtk
46	Loterías y Apuestas del Estado	2010-2021	Vídeo	https://www.youtube.com/watch?v=wqPnUrS2lDI
47	Centros Único	2010-2021	Vídeo	https://www.youtube.com/watch?v=QGF1yUdjIWE
48	Buitoni	2010-2021	Vídeo	https://www.youtube.com/watch?v=QUsIWuqYF-8
49	Eugin	2010-2021	Vídeo	https://www.youtube.com/watch?v=kFPikgGfVO4
50	Burger King	2010-2021	Vídeo	https://www.youtube.com/watch?v=UobfWf3V7V4
51	Correos	2010-2021	Vídeo	https://www.youtube.com/watch?v=9PG1OKXN5B8
52	Telepizza	2010-2021	Vídeo	https://www.youtube.com/watch?v=jYKOz5O9Xt4
53	Durex	2010-2021	Vídeo	https://www.youtube.com/watch?v=M8cJgKYqyso
54	Kas	2010-2021	Vídeo	https://www.youtube.com/watch?v=i4uX6_l4vzk
55	PSOE	2010-2021	Vídeo	https://www.youtube.com/watch?v=3Zj6Bp1XopE
56	Volkswagen Polo	2010-2021	Vídeo	https://www.youtube.com/watch?v=1Wg4M9uXEAA
57	Samsung	2010-2021	Vídeo	https://www.youtube.com/watch?v=oOJE4LHky4A
58	Santa Lucía	2010-2021	Vídeo	https://www.youtube.com/watch?v=9ZAQe_dqjbg
59	Correos	2010-2021	Vídeo	https://www.youtube.com/watch?v=92lFLgSzSP0
60	ViniGalicia	2010-2021	Vídeo	https://www.youtube.com/watch?v=Ewz-_cpnTTU
61	Intereconomía	2010-2021	Vídeo	https://www.youtube.com/watch?v=wfeBPOICFNM
62	HBO	2010-2021	Vídeo	https://www.youtube.com/watch?v=CVWhqpU4HKw
63	Orange	2010-2021	Vídeo	https://www.youtube.com/watch?v=2Lun491tv68

Source: Own elaboration using the collections and channels detailed.

Table 2 shows the list of advertisements analyzed in this research, including the name of the advertised brand, the decade of broadcast and the link to view the spot. Two researchers independently viewed the advertising directories and collections of advertisements described above to create the selection of 63 ads, and avoid bias. In cases where they did not agree on the choice of an advertisement, a third researcher made the decision on its suitability. However, this situation happened for two ads only.

## Results

In order to give a clearer structure to the results section, evidence extracted from the content analysis associated with each decade is presented. Firstly, Table 3 shows the most relevant historical and legislative facts.

**Table 3.** Relevant historical and legislative facts.

Decade	Relevant historical and legislative facts
**1960-1969.** **Franco's Dictatorship: 1936-1975/Starting point of the analysis was 1960 with the beginning of the broadcasting of Televisión Española.**	• (1936-1954) Article 431 of the Penal Code (referring to public scandal, and using “good manners” as a reference) applied, so there was a subjective interpretation of the law: there was or was not an offence depending on how scandalous the observer considered it to be. However, its treatment did not vary much from how it had been treated since the end of the 19th century. • 1936: Official Francoist condemnation of the poet and playwright Federico García Lorca. • (1954-1970) In 1954, Francoism approved a reform of the 1933 Law of Vagrants and Miscreants and began to explicitly criminalize homosexuality. A change was made to article 2.2, making it possible to declare "homosexuals, ruffians and pimps" to be "in a dangerous state" and to be subjected "to the security measures" of the law (BOE of 17 July 1954). This consideration of homosexuals as dangerous subjects led to an increase in the number of arrests, almost all of them men. • On 28 June 1969, following the police raid on the Stonewall Inn LGBT pub in New York and the revolt of some customers refusing to be arrested, the Pride movement was born (Snodgrass, 2019). From that moment on, Gay Pride Day has been celebrated internationally on that day in commemoration of this event. In Spain, due to the regime, the movement did not begin until later, but this historic milestone had an international influence on the fight for LGBT rights worldwide.
**1970-1979. End of the Dictatorship and first years of Democracy in Spain**	• 1970-1975 - Final stage of Francoism. Díaz (2019) The regulation of homosexuality evolved in the opposite direction to other transgressions: while it is considered that the Francoism of the 1970s began to become more flexible, in this area political control intensified, being one of the most condemned practices that did not enjoy a regulatory relaxation. Furthermore, and although the law does not explicitly allude to it, practice showed that it was not the sexual act itself that was punished, but men who broke gender roles and did not comply with the sacrament of marriage. In 1970 there was the new *Social Danger and Sexual Rehabilitation Act*, which was more paternalistic in nature: homosexuals were no longer punished, but those who "carry out homosexual practices", and the law was not intended to be punitive but re-educational (Mora-Gaspar, 2019). • 1973: The American Psychiatric Association eliminated homosexuality as a mental/psychological disorder by removing it from the *Diagnostic and Statistical Manual of Mental Disorders*, although this important milestone did not have a direct effect in Spain until the beginning of the 1990s. • Death of Franco, 1975. • 1978, adoption of Law 77/1978 of 26 December 1978, amending the Law on Dangerousness and Social Rehabilitation and its Regulations, when homosexuality was directly decriminalized. Some cases were still indirectly punished under the premise of "public scandal". This decriminalization, however, did not initially lead to greater social acceptance: it was felt that being LGBT should remain a private matter. • 1975-1980 – Beginning of democracy. Beginning of the social, cultural and political movement known as the Movida. Aparicio Cillán and Cimadevila Niño (2019, 17): " *Sex ceased to be taboo and many young people began to enjoy it naturally and without fear. Homosexuality and bisexuality became sexual orientations free of 'sin' and prison".* • Influence of the Movement on art, especially representative in the cinema. Two significant films were released in 1978: *El Diputado* by Eloy de la Iglesia and *Un hombre llamado Flor de Otoño* by Pedro Olea.
**1980-1989**	• Influence of the Movement on art. In music: songs of protest and with social claim such as *Ni tú ni nadie (*1984) by Alaska and *A quién le importa (*1986) by Alaska y Dinarama. The censorship barrier began to be overcome and homosexual protagonists began to be made visible.
**1990-1999**	• In the scientific field, the depathologization of same-sex attraction by the World Health Organisation took place on 17 May 1990. The idea that homosexuality and bisexuality are a mental disorder was eliminated. This day marks the International Day Against Homophobia, Transphobia and Biphobia. • In 1992, the Lesbian and Gay Collective of Cordoba was formed and became known by the acronym COLEGA.
**2000-2009**	• 2005: approval of same-sex marriage by 187 votes in favour, 147 against and 4 abstentions. Law 13/2005 of 1 July 2005, which amends the Civil Code on the right to marry (BOE of 2 July 2005), puts homosexuals and heterosexuals on an equal footing in terms of marriage rights, including the right to adoption. • The LGBT community and part of the population that accepts the normalization of this minority is rejoicing and celebrating at the same time that there are massive demonstrations called by conservative organisations against these social and legislative achievements for the gay and lesbian community. • In October 2006, the first monument dedicated to homosexuals in Spain was inaugurated in Sitges, an inverted pink triangle placed on the seafront. On 16 May 2009, the first monument dedicated to remembering the persecution of homosexuals during Franco's regime was inaugurated in Durango.
**2010-2021**	• In spite of the fact some people continued to oppose the evolution of an inclusive society in terms of the sexual freedom of the LGBT collective, the law 13/2005 was appealed against in the Constitutional Court. However, the Court dismissed the case of unconstitutionality filed in 2012 (Ruling 198/2012, 6 November 2012, published in the BOE, 2012). On 20 March 2011, a memorial “ in memory of gays, lesbians and transgender people who have suffered persecution and repression throughout history” is inaugurated in Barcelona, located in the Parc de la Ciutadella. • The open recognition of sexual status as a reason for non-discrimination in the public sphere – political, artistic, etc. – is taking place. On 16 December 2018, Ángela Ponce becomes the first transgender woman to compete in the Miss Universe competition.

The variables included in the content analysis lead us to present the evolution of advertising and the treatment of gay men and lesbians within it.

### Analysis associated with each decade

*1960-1969: Franco's dictatorship*

Gay men and lesbians are made invisible, being a taboo subject. In none of the advertisements viewed is there any allusion to the diversity of sexual orientations. The family model shown is univocal and traditional, with the exclusive representation of heterosexual couples. To a greater extent, young couples are shown, and depending on the product advertised, they are shown with or without children. Gender roles are usually shown in a differentiated way, which is congruent if we take into account the historical and cultural context: *Francoism.*

All the advertisements that were broadcast went through Franco's censorship, and no prejudices or stereotypes were broadcast through advertising. Therefore, in this decade there is a relationship between advertising treatment and the legislative and social treatment of gay men and lesbians, making them completely invisible.

*1970-1979: End of the dictatorship and early years of democracy in Spain*

Advertisements continue to perpetuate a great difference between the gender roles of men and women, either through their content or by the way in which their storyline is delivered. For example, the mother continues to be assigned the responsibility of childcare, and the father the ownership of objects such as cars (as seen in the Nesquik ad, No. 6). In addition, it is emphasized that products such as alcohol are consumed exclusively by men (as in the case of González Byass's Soberano Cognac, with the slogan “It's a man's thing”, ads Nos. 9 and 10). Particularly striking is the second González Byass ad (No. 10), which even shows a scene normalizing gender violence.

In this context, an univocal and traditional family and couple model is shown. Even in advertisements that claim to be inclusive, such as Coca Cola's (No. 8), the diversity of sexual orientations is ignored. Therefore, there is still a relationship between advertising treatment and the legislative and social treatment of gay men and lesbians.

*1980-1989: Movements and social changes*

Advertising in the 1980s experienced a process of modernization, in line with the rest of Spanish society.

As far as gender roles are concerned, there are ads in which a clear differentiation is evident (No. 11, by Monky; and No. 15, by Johnson), along with others in which they appear practically undifferentiated (No. 13, by Riera-Marsá; and No. 14, by Princesa). In the latter, a greater co-responsibility of men in domestic tasks can be observed compared to previous decades (No. 13). However, it is true that sometimes these men are still presented with a humorous and ridiculing tone, as if they are facing impossible tasks to perform (this is the case with No. 12, Scotch Brite).

In addition to all of the above, there is a greater normalization of sexuality and eroticism. For this purpose, puns and double meanings are used (advertisement No. 14, from Galletas Princesa). However, for the time being, the family model seems to remain unique and the couples represented are heterosexual and young.

We should remember that, in the 1980s, the process of protesting to demand rights and equality between heterosexuals and gay men and lesbians continued. The World Health Organization still considered gay men and lesbians “sick”; therefore, there was still no effective integration into society. In other words, there was still a relationship between the legal and sociocultural context and the reality reflected in advertisements.

*1990-1999: Social openness and the evolution of equality*

It should be noted that, as far as sexuality is concerned, the path of openness started in the previous decade continues. Usually, double meanings are still used when referring to this type of topic (No. 16, from Starlux; and No. 19, from Rumba Total), and advertisers manage to connect these ambiguities with the promotion of the product (for example, a woman with her partner saying “this one does it the way I like it”, to promote the Starlux granulated broth).

During this decade, equality between gender roles began to be reflected more effectively. Men appear more frequently as jointly responsible for household chores and childcare (advertisements Nos. 16 and 18).

In addition, announcements begin to be made that little by little highlight the uniqueness and independence of being women. Ad No. 20, from *Woman* magazine, is a good example. Although this modernization was rather lax, it opened the door for women to want to feel beautiful in themselves, not for their husbands (the attitude changes regarding submission, from advertisements such as No. 1) and, the most interesting thing in our field study, opened the door to women who do not like men.

In the same way, it is worth noting the clear contrast in children's social relations between the advertisements of the 1960s/1970s and those made in the 1990s: in the former, boys and girls are shown playing separately, in different groups and with different toys (spots No. 2 and No.7), while in the latter they are shown playing together and cooperating (adverts Nos. 3 and 17).

The evolution in the concept of family and love follows the same lines as the evolution in gender roles: a lax openness. A representative case is No. 18, in which the Renault Laguna automobile is promoted and a father appears as the sole caregiver for his son, without a partner; therefore, it could favour selective perception by the viewer to consider him as a gay man, heterosexual or bisexual. However, given the social context in which it is inserted, the advertiser's intention was probably simply to awaken the patriarchal protective instinct.

Finally, the ad for the compilation album *Rumba Total* deserves a special mention. Although advertising about the transsexual collective is not the subject of this research, ad No. 19 is probably one of the first to allude to the LGBTIQ+ community in a clear way. In it, Cristina “La Veneno”, a popular transgender woman of the 1990s, is presented as the protagonist, and a play on words is made between the pack of songs offered and her genitalia, recreating the famous leg-crossing scene from the movie *Basic Instinct.*

Taking into account the above and that during this decade the depathologization of gay men and lesbianism took place, we can conclude that during the decade there was also a relationship between social advances and advertising treatment.

*2000-2009: Normalization and acceptance at the social and legislative levels*

With the arrival of the new millennium, the normalization of gender and sexual diversity in advertising is consolidated. For example, sexuality and nudity began to be shown more explicitly, without the need to use ambiguities as in previous decades. The approval of same-sex marriage coincided with a greater normalization, in advertising terms, of the imagery related to the collective.

As far as gender roles are concerned, the co-responsibility of household chores is fully achieved as far as their representation in advertising is concerned, going beyond the “aesthetic chores on camera” (cooking and taking care of the children, basically): one example, among others, is Bosch ad No. 22, which shows a man doing the ironing. In addition, equality is starting to move more in the direction of femvertising. Nevertheless, ads such as Heineken's (No. 24) continue to depict differences by gender: girls are interested in clothes and boys interested in beer.

**Figure 1.**
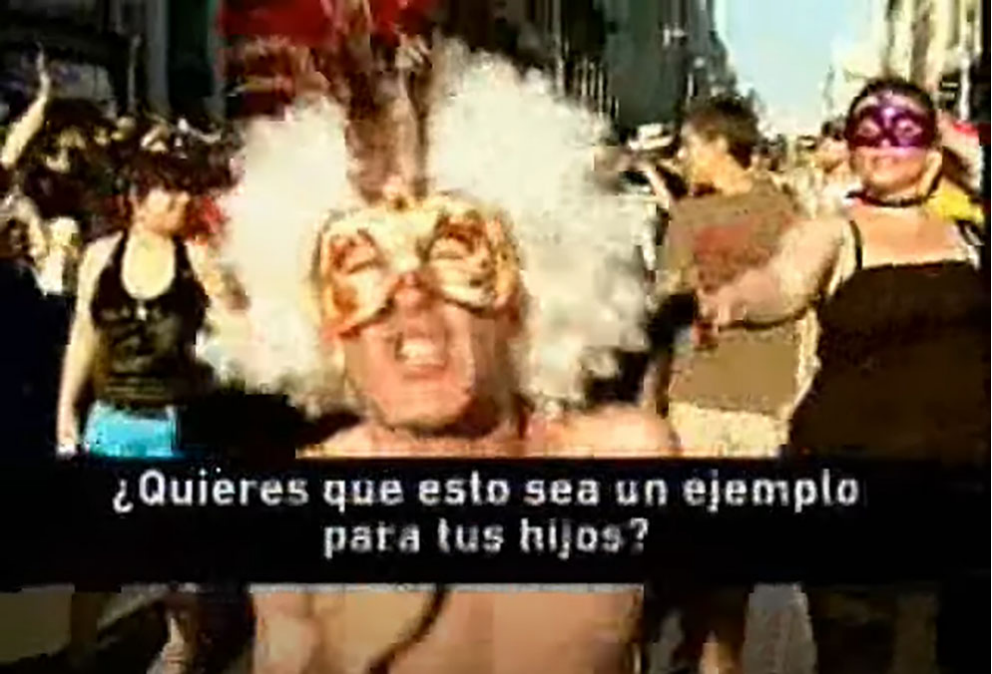
An advertisement broadcast on Intereconomía TV channel on the occasion of Gay Pride, taken from a blog site online. (Original source: *Las Malas Lenguas* ( Intereconomia TV, 2009).) Translated text from the ad: Do you want this to be an example for your children?.

These are turbulent times for reflecting family diversity in advertising. In a very clear way, there coexists an advertising that reflects the most traditional society and another “more transgressive” one that represents the social evolution regarding the gay men and lesbian collective. Thus, we find advertisements that, even though they are clearly endeavouring to reflect the variety of couples in love, as they see it, do not show gay and lesbian couples (advertisement No. 23, IKEA). Others directly sexualize relationships between women and objectify them (ad No. 21, *Musicón del Verano*). In the most extreme case, we find companies such as the communication group *Intereconomia* (No. 25) that punish the demands of the rights of the collective, using an advertising style that tries to instill “fear” ( Figure 1). That is to say, some advertisements are responsible for reflecting the discontent generated in part of the population by the approval of same-sex marriage.

However, the majority do support an inclusive and diverse society. Therefore, in this instance, according to the spots in our sample there is not a complete relationship between advertising’s treatment of the gay men and lesbian community and the legislative framework.

*2010-2021: Current decade*

The period 2010-2021 sees a notable increase in the number of appearances of gay men and lesbian couples and individuals in audiovisual advertising. However, although the growth is considerable compared to previous decades, the percentage with respect to the total number of advertisements (of a commercial nature) is still not very significant.

Based on the content analysis, Table 4 shows the congruence between historical contexts and advertising related to the gay men and lesbian community.

**Table 4.** Congruence between historical context and the treatment of the LGTB community in advertising (%).

		It is congruent	It is not congruent
Historical context	1960-2009	92	8
	2010-2021	97	3

**Figure 2.**
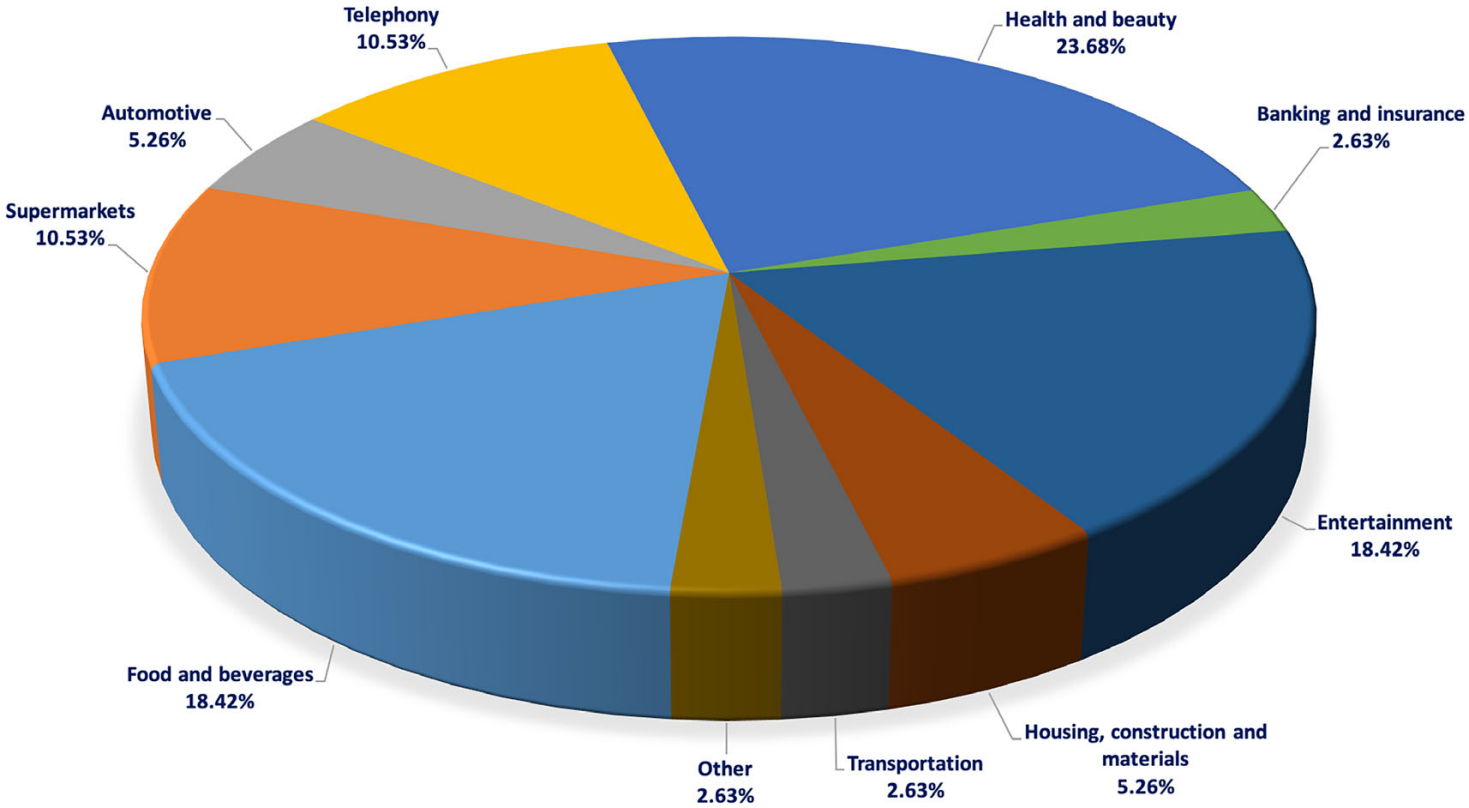
Weighting of each sector in the sample of advertisements for the years 2010-2021. Source: Own elaboration.

First, it is worth noting the wide variety of products that include depictions of the gay men and lesbian collective. We find everything from dating apps to soft drinks and cleaning products; naturally, this diversity is reflected in the wide range of sectors present in the sample ( Figure 2). The sector that includes the most depictions of the gay men and lesbian community is beauty and hygiene (23.7%), followed by food and entertainment (18.42% each).

**Figure 3.**
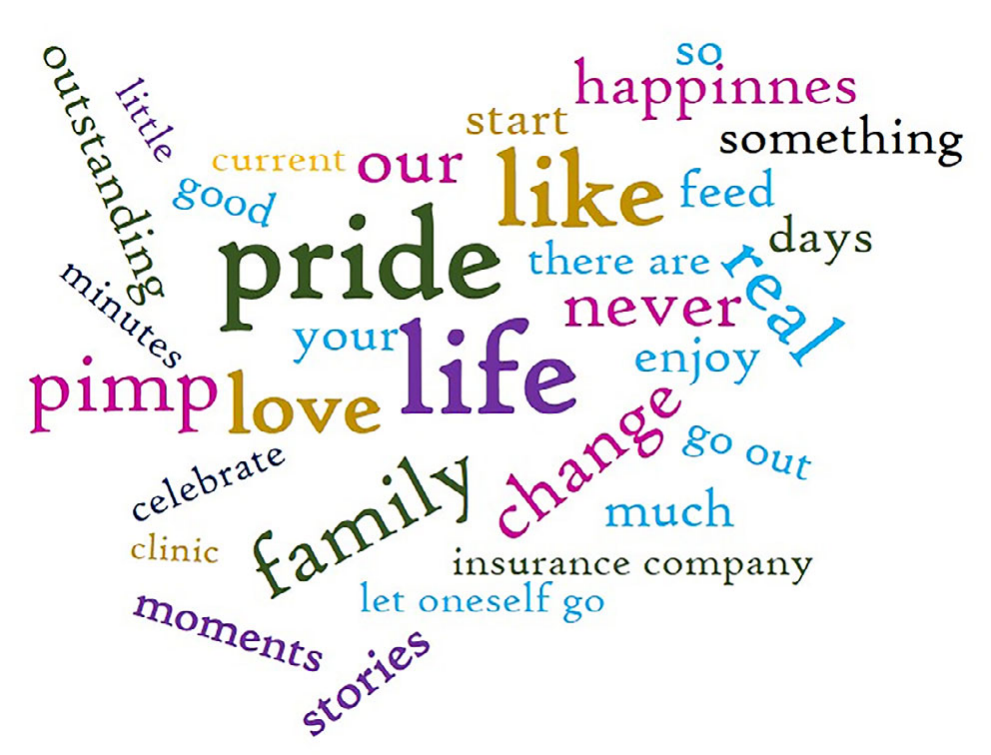
Wordcloud of the main words used in the slogans. Source: Own elaboration.

The research on slogans shows that reference is made to the gay men and lesbian collective depending on the tone of the advertisement. That is to say, if it has an educational or vindicatory function (as in the case of Burger King's ad No. 50 and La Sexta TV channel's ad No. 43), words that aim to empower the collective, such as “pride”, are usually included. The use of this type of slogan tends to have a seasonal character, being especially frequent during the LGBTIQ+ Pride celebration (end of June). However, there is another group of ads that simply integrate and normalize gay men and lesbian protagonists within the story told in the ad (for example, No. 28), so the slogan is usually related to the product ( Figure 3).

Regarding the relationship between the representations of the gay men and lesbian community in the advertising spots and the fact that both were the target audience of the brands, we observed that gay men and lesbian couples and individuals are reflected in advertising regardless of whether or not they are the target. Specifically, only in 2 of the 38 cases that show situations or environments related to the collective, is sexuality a relevant segmentation criterion (spots Nos. 49 and 53). The rest are products aimed at the general public, of which gay men and lesbians are only a part.

On the other hand, the variable “explicitness of the message”, that is, the way in which the reference to the gay men and lesbian community is produced (whether directly or through ambiguities and double meanings) indicates that 71% of the ads in the sample do so explicitly. The words and expressions exchanged by the partners (dialogues showing mutual affection, use of romantic appellatives such as “sweetheart”, etc.) as well as the images (the way they look at each other, hug and kiss, for example) are the instrument for this. The remaining 29% of ads show the relationship between the protagonists in an ambiguous way. The main motivation to provoke this ambiguity is probably to favour a more selective perception, i.e. that the viewer is the one who assigns the label of “friends” or “couple” according to his or her reality. This issue leads us to evidence that the relationship between the characters is often ambiguous (No. 27, No. 48 and No. 54).

**Figure 4.**
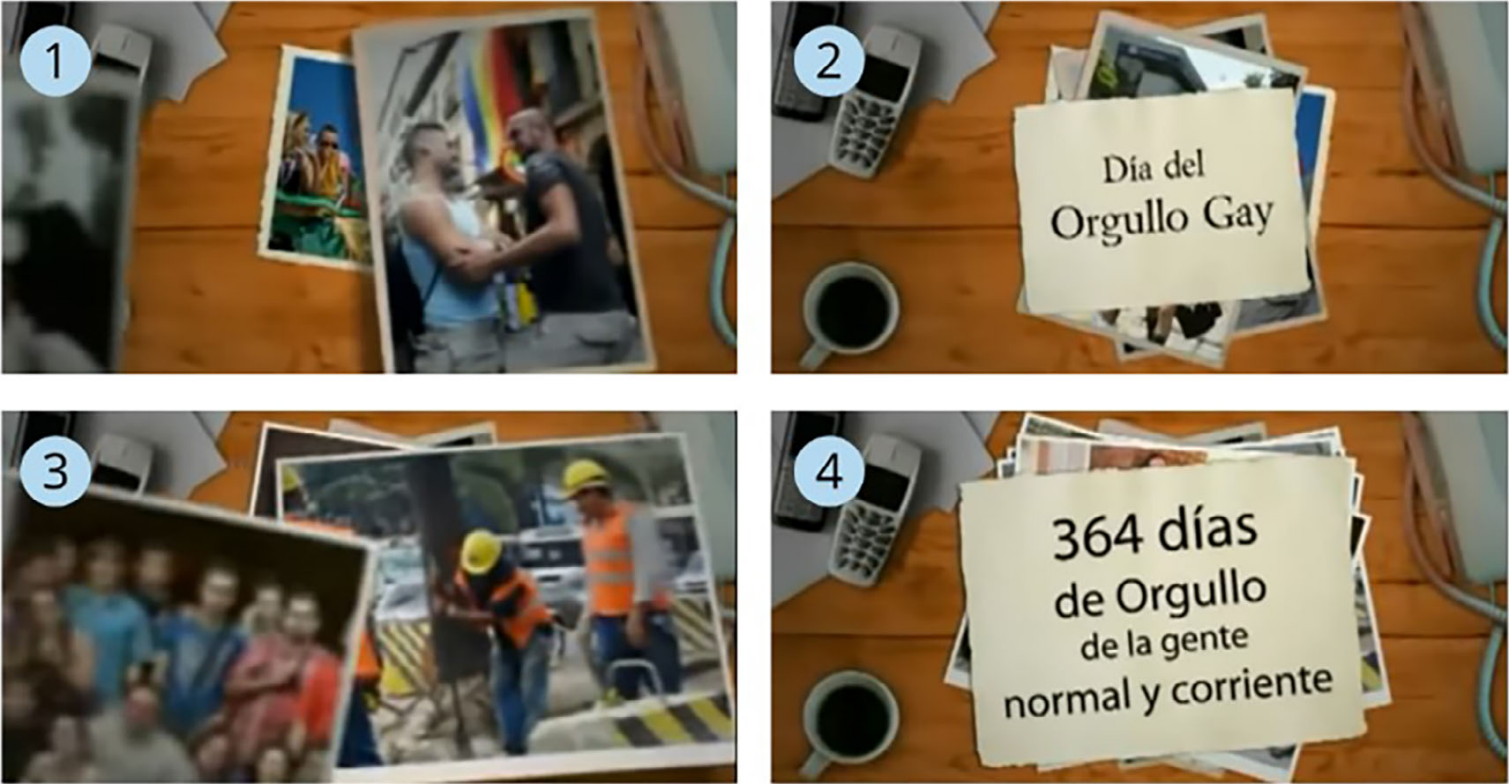
Sequence of stills from commercial No. 61, broadcast on *Intereconomía TV.* Translated text from the ad: Image 2: Gay Pride Day; Image 4: 364 days of Pride for ordinary people.

As for stereotypes, it is worth remembering that the three ways of expression are the storyline way (reproducing clichés about gay men and lesbians, such as promiscuity), the visual way (reproducing clichés about their clothing, appearance, gestures, etc) and the sound way (stereotypes about the way they speak and express themselves). The results show that we have gone from an almost total invisibilization in previous decades, to an inclusion of the collective made from respect, and with the absence of these clichés. Thus, the ads from this decade reflect a diverse family model. However, there is a discriminatory advertisement (spot No. 61), but it is an isolated and specific case ( Figure 4), therefore, the gay men and lesbian community as a sexual and amorous option ends up becoming visible and normalized.

**Figure 5.**
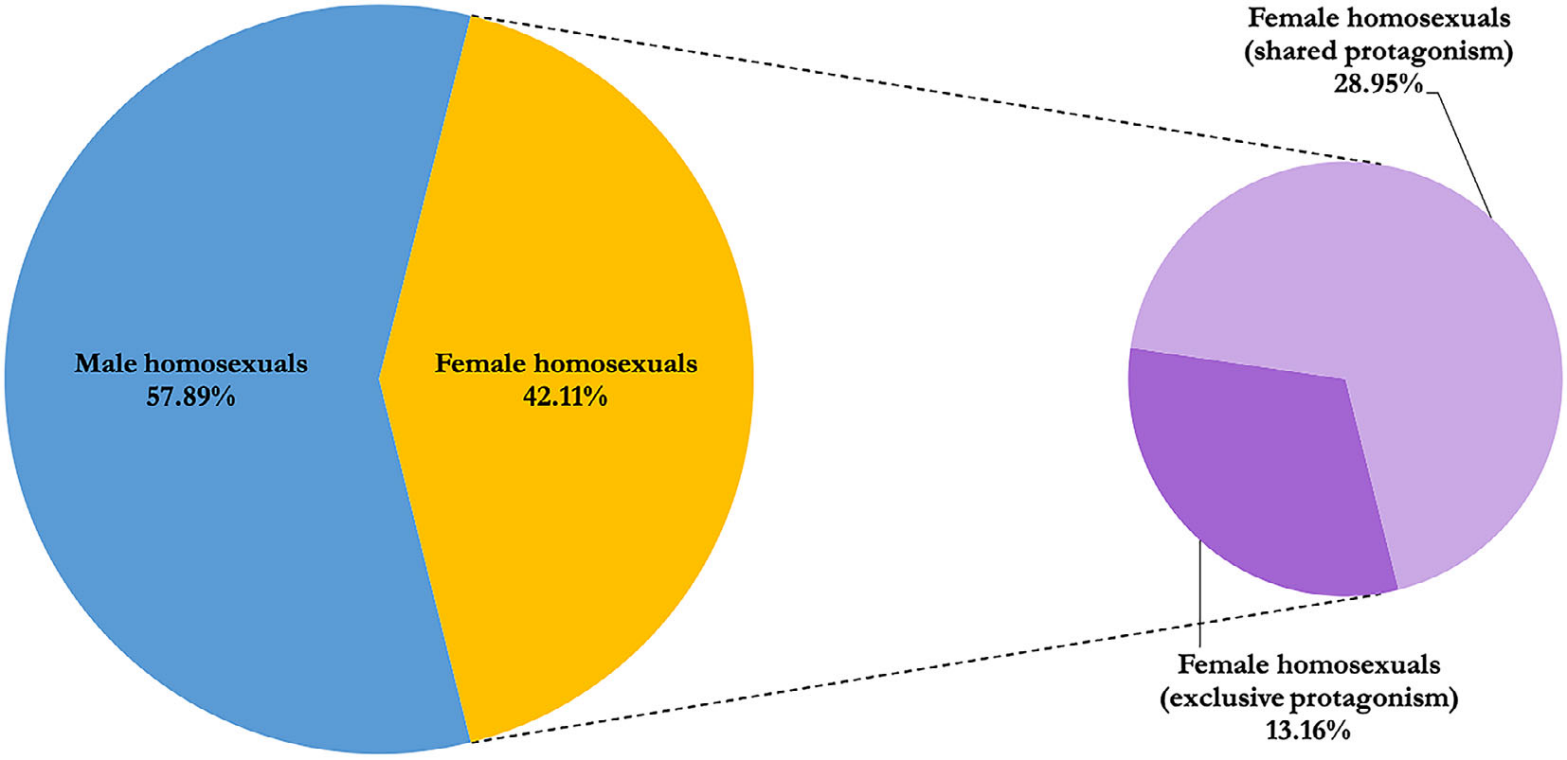
Representation of gay men and lesbian´ couples. Source: Own elaboration.

Regarding the types of couples represented, it is worth mentioning that we find a great diversity of ways of showing them: while in some spots only one typology is shown (for example, a gay couple), in others the range is wider and more diversity is shown (for example, gay men and lesbians and heterosexuals being co-protagonists). Specifically, in 47.37% of the ads, gay men and lesbian couples share space with heterosexual couples ( Figure 5).

In the same line, the distribution of percentages of the representation of traditional family models in the decades studied gives a concrete view on the evolution of openness to amorous diversity in the last targeted period (2010-2021). The traditional family and couple model is represented in 68% of ads while the diverse family and couple models appear only in 16% of ads in the years from 1960 to 2009. It should be emphasized that 16% of advertisements without a clear model are included. By contrast, the most contemporary era (2010-2021) evokes diverse family and couple models in 100% of cases.

Another noteworthy variable where there are contrasting data between decades is gender roles. First, the roles are well differentiated (totally or sufficiently) in earlier ads while in recent years, advertisers opt for portraying no differences in most of the cases ( Table 5).

**Table 5.** Representation of gender roles.

		Totally differentiated	Sufficiently differentiated ^1^	Neutral	Few differences	No differences
Gender roles	**1960-2009**	28	24	16	4	28
	**2010-2021**	0	0	0	3	97

^1^ Gender roles were measured with a Likert scale with 5 points. No. 5, “Totally differentiated”, means the gender roles of characters in ads follow the traditional pattern of women and men in society.

It can be determined that in the last decade analyzed, advertising is mostly related to social and legal changes in Spain in terms of affectionate relationships. Thus, a respectful inclusion of the gay men and lesbians collective is made, trying to reflect an increasingly common social reality, so that brands can address any type of consumer regardless of their sexual orientation.

## Discussion

This research work provides new insights about how advertising and society interplay and feed each other. Although this assertion was already established by Pollay (1986) as well as in more recent works such as those by Schmidt and Stocker (2013) and McDonald *et al.* (2020), the focus on sexual and amorous diversity evidences how companies continuously seek a balance in their advertising between the reflection of what society is, with its stereotypes and strongest beliefs, and the changes/advances that are inevitably generated within it.

The analysis of advertising content and the historical review leads us to evidence the close relationship of society, legislation and advertising. This struggle in the creative strategy responds to the need to connect with the target audience, being cautious with the possible reticence and rejection of a part of it but being consistent with representation in its imagery of minorities, who interestingly commercially crave to be reflected. In this regard, the findings coincide to some extent with the research works of Akermanidis and Venter (2014), Hetsroni (2011), Chae *et al.* (2016), Akestam *et al.*, (2017), Iribure Rodrigues (2017), Holz Ivory (2019), Read *et al.* (2019), Madinga *et al.* (2020) and Cheah *et al.* (2021). At the same time, it coincides with what is indicated by Taylor and Costello (2017) – advertising that includes through images or messages the targeted topics is designed to express awareness and the corporate social responsibility of companies.

The analysis of advertising in Spain over six decades, since the beginning of television in Spain, shows a progressive increase in the representation of the gay and lesbian collective, as do other investigations where a historical review of the presence of this group in advertising is undertaken ( Branchik & O’Leary, 2016; Descubes *et al.*, 2018; Iribure Rodrigues & Gallina Zanin, 2014; McDonald *et al.*, 2020; Ragusa, 2005). Nevertheless, the percentage of ads in which the gay men and lesbian imagery is explicitly or implicitly present remains proportionally low over the total volume of television spots analyzed.

The research evidence shows how it is common to use double meanings or the use of messages and/or images that can be interpreted openly to connect with one or another audience, as pointed out by the Social Identity Theory proposed by Taylor and Costello (1985). This common behaviour by the brands analyzed in recent decades coincides with Read *et al.* (2019), Ruggs *et al.* (2018) and Gong (2020), who emphasize the existence of what they call “mixed sexual ads”, i.e., spots in which the representation of gay men and lesbian and heterosexual themes is ambiguous, undertaken to address some without risking the arousal of sensitivities if there is a section of the viewers reluctant to accept the former.

Attention has been drawn to an issue worthy of more detailed reflection in future research. Coinciding with previous research, such as that of Ragusa (2005), there is greater coverage of gay men than of lesbians in the advertising imagery. This is a fact that contrasts with the findings of studies that conclude that the heterosexual population shows greater acceptance/less criticism of images depicting relationships between two people of the same gender if they are women than if they are men ( Descubes *et al.*, 2018; Madinga *et al.*, 2020).

Based on the above, the research questions can be answered by showing that there has been a positive change, consistent with the times, in the advertising imagery and its treatment of gay men and lesbians since the 1960s in Spain (RQ1). On the other hand, the representations seem to have a significant relationship with the major milestones and legislation in this field (RQ2), although it can be seen that this evolution is taking place cautiously and coexisting with more classical representations. Finally, love and sexual diversity is more openly expressed, but with a great deal of recourse to interpretable messages or images in order to connect with one or another target according to their identity. Thus, the stance adopted in advertising continues to balance between the disruptive and the classic (RQ3).

The research goes a step further by showing that advertising and the times are moving forward hand in hand, favouring a cautious change that ends up adapting to the most disruptive demands of a part of society. To this end, stigmas, stereotypes or clichés associated with gay men and lesbians are being blurred and inclusive advertising is being projected. In the most recent years of the analysis, discriminatory messages are hardly detected, curiously in a lower proportion for Spain than in the work of McDonald *et al.* (2020), focused on American advertising. The research considers the visualization itself in adverts as a step forward that once again emphasizes the social function of advertising. Consequently, the contribution of this work lies not only in its impact on the analysis of advertising imagery, since it also introduces a theoretical contribution that finds its parallel in the term femvertising used by Bahadur (2014): Queervertising. This concept underlines the adaptation of brands to the social, political and economic changes of the LGBTIQ+ community, as well as their contribution to its empowerment, visualization and inclusive recognition in society.

## Conclusions

Advertising reflects society, as well as supports new movements or relevant changes that are forged within it. In this sense, the contribution of the concepts of gender and sexual diversity in advertising represents a new prism through which to advance in studies on the social function of advertising, and on being inclusive for historically invisible and stigmatized minority groups. Advertising thus becomes a mainstreaming and empowerment tool for this minority. Sexual and amorous diversity is progressively normalized in advertising creative axes, although the volume of these representations is not so significant in the total number of minutes of television advertising achieved.

Likewise, the effort of companies to include messages that connect with gay men and lesbians in their advertising, but without being excessively explicit or transgressive in order to avoid possible rejection by part of the audience reluctant to accept gay men and lesbian imagery, is underlined. The interaction between society, through the review of key milestones and Spanish legislation for this collective, and advertising seems to show a significant relationship. Based on the above, it can be affirmed that advertising favours changes and social evolution through its commercial messages, both as a means of connecting with the LGBTIQ+ community and to develop its corporate social responsibility and position itself as a gay-friendly communicative tool.

### Limitations and future research lines

There is no doubt that in future years, Queervertising will be a fertile area in terms of research. The main limitation of this work is the difficulty in establishing a determinant cause-effect relationship between legislation, movements and social milestones linked to gay men and lesbians and their projection in advertising imagery. However, the proposed methodology has evidenced solid relationships between these dimensions and clarifies the evolution over time of the inclusion of gay men and lesbians in advertising, as well as significant changes in the treatment of this group.

This work has had to focus on gay men and lesbians without it being possible to consider other groups such as transsexuals or bisexuals due to the lack of written or audio text, symbolism or images of them in the advertising under analysis. Therefore, it is presented as a future line of research of interest to complete the vision of the phenomenon studied.

Likewise, it would be useful to make an international comparison incorporating other European countries or even contrasting the results with countries with less legislative progress and recognition of the rights of the LGBTIQ+ community.

Despite the usefulness of content analysis, a final future line of research would be to triangulate the data to include the perception and motivations of three main groups. Firstly, by approaching companies directly through a focus group or in-depth interviews. Likewise, it would be interesting to know how advertising copywriters take on this challenge of inclusive society in advertisements. Finally, by involving gay men and lesbians and heterosexual audiences in the fieldwork with the aim of delving into and comparing their perceptions and feelings regarding ads.

### Practical implications

Corporate advertising changes and evolves along with society. The most relevant practical implication would be to make brand owners aware of the suitability of exercising their social function in a more disruptive and less cautious way, becoming agents promoting changes and innovative perspectives in society and positively relating their brands as gay-friendly. Urging advertisers and researchers to work for a fairer and more inclusive society through their messages is a complex and ambitious, but unavoidable and necessary challenge.

## Data Availability

As detailed, secondary data for analysis were obtained from the Spanish Association of Advertisers (Asociación Española de Anunciantes, AEA); and from complementary databases provided by *the El Publicista* journal ( El Publicista, 2021) and the Sanz Channel ( Sanz, 2016). We have provided the data produced by our content analysis of advertisements, using the analysis sheet in Table 1, at Figshare; ‘Data – Queervertising – reviewed’ is at: https://doi.org./10.6084/m9.figshare.21953030.v1 ( Iglesias-Sánchez, 2023). The Figshare project contains the following underlying data:
– Data-Queervertising-reviewed.xlsx Data are provided under the terms of the Creative Commons Attribution 4.0 International License (CC-BY 4.0).

## References

[ref1] Abrams MH : *The mirror and the lamp: Romantic theory and the critical tradition.* Oxford University Press;1953

[ref2] Akermanidis E Venter M : Erasing the line between homosexual and heterosexual advertising: A perspective from the educated youth population. *The Retail and Marketing Review.* 2014;10(1):50–64.

[ref3] Åkestam N Rosengren S Dahlen M : Think about it – can portrayals of homosexuality in advertising prime consumer-perceived social connectedness and empathy? *Eur. J. Mark.* 2017;51(1):82–98. 10.1108/EJM-11-2015-0765

[ref4] Angelini JR Bradley SD : Homosexual Imagery in Print Advertisements: Attended, Remembered, but Disliked. *J. Homosex.* 2010;57(4):485–502. 10.1080/00918361003608665 20391007

[ref5] Bahadur N : *Femvertising ads are empowering women - and making money for brands.* Huffington Post;2014. Reference Source

[ref6] Bond BJ Farrell JR : Consumer Responses to Print Advertisements Featuring Gay Males Over Time. *Sex. Cult.* 2020;24(5):1432–1442. 10.1007/s12119-019-09692-6

[ref7] Branchik BJ O’Leary B : Funny, scary, dead. *J. Hist. Res. Mark.* 2016;8(4):524–544. 10.1108/JHRM-07-2015-0027

[ref8] Chae Y Kim Y Johnson KKP : Fashion brands and gay/lesbian-inclusive advertising in the USA. *Fashion, Style & Popular Culture.* 2016;3(2):251–267. 10.1386/fspc.3.2.251_1

[ref9] Cheah Teah M Lee S : Straight eye for the queer ad: Attitudes, skepticism, inferences of manipulative intent and willingness to buy. *Asia Pac. J. Mark. Logist.* 2021;33(5):1220–1238. 10.1108/APJML-03-2020-0124

[ref10] Cunningham GB Melton N : Signals and Cues: LGBT Inclusive Advertising and Consumer Attraction. *Sport Mark. Q.* 2014;23:37–46.

[ref11] Descubes I McNamara T Bryson D : Lesbians’ assessments of gay advertising in France: Not necessarily a case of ‘La Vie en Rose?’ *J. Mark. Manag.* 2018;34(7–8):639–663. 10.1080/0267257X.2018.1474242

[ref12] El Publicista: *YouTube Channel specialised in advertising communication and marketing.* El Publicista;2021. Reference Source

[ref13] Feng J Wu DD : Changing ideologies and advertising discourses in China: A case study of Nanfang Daily. *J. Asian Pac. Commun.* 2009;19(2):218–238. John Benjamins. 10.1075/japc.19.2.06fen

[ref14] Fowler K Thomas V : A content analysis of male roles in television advertising: Do traditional roles still hold? *J. Mark. Commun.* 2015;21(5):356–371. 10.1080/13527266.2013.775178

[ref15] Gong ZH : Crafting Mixed Sexual Advertisements for Mainstream Media: Examining the Impact of Homosexual and Heterosexual Imagery Inclusion on Advertising Effectiveness. *J. Homosex.* 2020;67(7):916–939. 10.1080/00918369.2018.1564005 30633658

[ref16] Grau SL Zotos YC : Gender stereotypes in advertising: A review of current research. *Int. J. Advert.* 2016;35(5):761–770. 10.1080/02650487.2016.1203556

[ref17] Hetsroni A : Pluralistic media ignorance: Presence and causes. *Soc. Sci. J.* 2011;48(2):324–334. 10.1016/j.soscij.2010.12.004

[ref18] Holbrook MB : Mirror, Mirror, on the Wall, What’s Unfair in the Reflections on Advertising? *J. Mark.* 1987;51(3):95–103. JSTOR. 10.2307/1251650

[ref19] Holz Ivory A : Sexual Orientation as a Peripheral Cue in Advertising: Effects of Models’ Sexual Orientation, Argument Strength, and Involvement on Responses to Magazine Ads. *J. Homosex.* 2019;66(1):31–59. 10.1080/00918369.2017.1391558 29023207

[ref47] Iglesias-Sánchez P : DATA- Queervertising-reviewed.xlsx. figshare.Dataset.2023. 10.6084/m9.figshare.21953030.v1

[ref21] Intereconomia TV: Dia del Orgullo Gay [Advertising]. 2009. Reference Source

[ref22] Iribure Rodrigues A : Entre as representações e as repercussões das homossexualidades: Uma análise da publicidade veiculada na TV aberta e seus desdobramentos na rede social. *Conexao - Comunicaçao e Cultura.* 2017;16(32):135–155. 10.18226/21782687.v16.n32.06

[ref23] Iribure Rodrigues A Gallina Zanin V : As representações das homossexualidades em anúncios veiculados na televisão brasileira entre os anos de 2008 e 2012. *Conexao - Comunicaçao e Cultura.* 2014;13(25):99–119.

[ref24] Jacob L : *Americans are twice as likely as Europeans to identify as LGBTQ.* Dalia Research;2017. Reference Source

[ref25] Krippendorff K : *Content Analysis: An Introduction to its Methodology.* 2nd ed. Sage Publications, Inc.;2004.

[ref26] Madinga NW Broster P Kappatos A : Exploring heterosexual responses to lesbian and gay-themed advertisements in South Africa. *Communitas.* 2020;25:1–20. 10.18820/24150525/Comm.v25.8

[ref27] McDonald RE Laverie DA Manis KT : The Interplay between Advertising and Society: An Historical Analysis. *J. Macromark.* 2020;41:585–609. 10.1177/0276146720964324

[ref28] Mikkonen I : Negotiating subcultural authenticity through interpretation of mainstream advertising. *Int. J. Advert.* 2010;29(2):303–326. 10.2501/S0265048710201166

[ref29] Molina Rodríguez-Navas P Simelio Solà N Ibarz Gelabert J : Televisión, cine y publicidad, fuentes de conocimiento del pasado y del presente. *Historia y Comunicación Social.* 2014;18:461–471. 10.5209/rev_HICS.2013.v18.44342

[ref30] Oakenfull GK Greenlee TB : Queer eye for a gay guy: Using market-specific symbols in advertising to attract gay consumers without alienating the mainstream. *Psychol. Mark.* 2005;22(5):421–439. 10.1002/mar.20066

[ref31] Oakenfull GK Mccarthy MS Greenlee TB : Targeting a Minority without Alienating the Majority: Advertising to Gays and Lesbians in Mainstream Media. *J. Advert. Res.* 2008;48(2):191–198. 10.2501/S0021849908080239

[ref32] Olivares-Delgado F Iglesias-Sánchez PP Benlloch-Osuna MT : Resilience and Anti-Stress during COVID-19 Isolation in Spain: An Analysis through Audiovisual Spots. *Int. J. Environ. Res. Public Health.* 2020;17(23). 10.3390/ijerph17238876 33260338 PMC7730842

[ref33] Orr R Van Rheede Van Oudtshoorn GP Kotzé TG : The perceptions of consumers aged 18-30 of “lesbian” appeals in advertising. *Communicare: J. Commun. Sci. S. Afr.* 2005;21(1):49–68.

[ref34] Pollay RW : The Distorted Mirror: Reflections on the Unintended Consequences of Advertising. *J. Mark.* 1986;50(2):18–36. JSTOR. 10.2307/1251597

[ref35] Poushter J Kent N : *The Global Divide on Homosexuality Persists.* Pew Research Center;2020. Reference Source

[ref36] Ragusa AT : Social change and the corporate construction of gay markets in the New York Times’ advertising business news. *Media Cult. Soc.* 2005;27(5):653–676. 10.1177/0163443705055721

[ref37] Read GL Innis IJ Driel II van : Mates or Married? Implications of Gender Composition and Physical Intimacy on Evaluation of Images Tested for Advertising. *Commun. Res. Rep.* 2019;36(3):220–230. 10.1080/08824096.2019.1605894

[ref38] Ruggs EN Stuart JA Yang LW : The effect of traditionally marginalized groups in advertising on consumer response. *Mark. Lett.* 2018;29(3):319–335. 10.1007/s11002-018-9468-3

[ref39] Sanz C : *Playlist TV commercials in Spain.* Anuncios de TV de España;2016. Reference Source

[ref40] Schmidt S Stocker P : Omunicação, juventude e diversidade. *Revista Eletrônica Internacional de Economia Política Da Informação, Da Comunicação e Da Cultura.* 2013;15(3):178–189.

[ref41] Shinoda LM Veludo-de-Oliveira T Pereira I : Beyond gender stereotypes: The missing women in print advertising. *Int. J. Advert.* 2021;40(4):629–656. 10.1080/02650487.2020.1820206

[ref42] Taylor CR Costello JP : The Social Identity Theory of Group Behavior. *PsychoIogy of Intergroup Relations.* Austin:1985; (Vol.2, pp.7–24).

[ref43] Taylor CR Costello JP : Corporate Social Responsibility and the Portrayal of Minority Groups in Advertising. *Handbook of Integrated CSR Communication.* Springer;2017; (pp.361–375). 10.1007/978-3-319-44700-1_20

[ref44] Um N-H : Does gay-themed advertising haunt your brand? *Int. J. Advert.* 2014;33(4):811–832. 10.2501/IJA-33-4-811-832

[ref45] Yu T-F : Class as a method to localise queer studies in Hong Kong. *Gend. Place Cult.* 2018;25(2):309–312. 10.1080/0966369X.2017.1407298

[ref46] Zmuda N : *Ad campaigns are finally reflecting diversity of U.S.* AdAge;2014. Reference Source

