# Supplementary material for: Queervertising: An empowerment tool for the gay men and lesbian community
Source: F1000Res. 2023 Mar 17;12:295. [Version 1] doi: 10.12688/f1000research.126882.1 (PMC10036954; doi:10.12688/f1000research.126882.1)
Supplement: Peer review history

# Peer review: Reviewer response for version 1

**DOI:** 10.5256/f1000research.139338.r167056

**Published:** 2023-04-05

**Authors:** Irene García Medina

**Affiliations:** 1 Glasgow Caledonian University, Glasgow, Scotland, UK

The article analyses how advertising and society interact with the gay and lesbian community and all the changes that happened in Spain from 1960 until 2021.

 The methodology is adequate (a content analysis and a review of legislation and historical milestones).

 The findings and conclusion are correct and the references used are good, both in number and in importance.

 For all this, I consider this article of a very good quality and very important for the society and the academia.

Is the work clearly and accurately presented and does it cite the current literature?

Yes

If applicable, is the statistical analysis and its interpretation appropriate?

Yes

Are all the source data underlying the results available to ensure full reproducibility?

Yes

Is the study design appropriate and is the work technically sound?

Yes

Are the conclusions drawn adequately supported by the results?

Yes

Are sufficient details of methods and analysis provided to allow replication by others?

Yes

Reviewer Expertise:

Marketing, Advertising

I confirm that I have read this submission and believe that I have an appropriate level of expertise to confirm that it is of an acceptable scientific standard.

# Peer review: Reviewer response for version 1

**DOI:** 10.5256/f1000research.139338.r167055

**Published:** 2023-03-23

**Authors:** Pedro A. Pereira Correia

**Affiliations:** 1 University of Madeira, Funchal, Portugal

This is a very original and interesting topical research study with a historic approach focused on Spain. I often read and contribute to studies related to gender image in advertising but it is not so usually the analysis of LGBT and other “special collective”. Thus, thank you for the opportunity to review this essay. I wish the author(s) very well as they continue their important work.

 The list of features used for the content analysis is very comprehensive and could be used to analyze ads in other contexts, specially providing international comparison. The paper is very well written and the findings are quite interesting. 

 In terms of improvement, I have the following suggestions:

- In the abstract, the researchers mention that the goal of the ads has moved from a commercial angle to a more social function, however, this is not really emphasized in the discussion in contrast with the mentioned Mirror Theory. The theoretical implications could be interesting.
- In a future line of research, could gender-fluid and other trends be included?

Is the work clearly and accurately presented and does it cite the current literature?

Yes

If applicable, is the statistical analysis and its interpretation appropriate?

Yes

Are all the source data underlying the results available to ensure full reproducibility?

Yes

Is the study design appropriate and is the work technically sound?

Yes

Are the conclusions drawn adequately supported by the results?

Yes

Are sufficient details of methods and analysis provided to allow replication by others?

Yes

Reviewer Expertise:

Marketing and communication

I confirm that I have read this submission and believe that I have an appropriate level of expertise to confirm that it is of an acceptable scientific standard.

# Peer review: Reviewer response for version 1

**DOI:** 10.5256/f1000research.139338.r167054

**Published:** 2023-03-22

**Authors:** Rafael Ravina-Ripoll

**Affiliations:** 1 Business Organization Department and INDESS, Faculty of Economics and Business, University of Cadiz, Cádiz, Spain

I have enjoyed reading this paper. I appreciate that the author(s) focus on this issue: representations of gay men and lesbians collective is original and infrequent in literature. I appreciate the effort made to analyse how these collective are represented in ads. I believe research around this topic would be of interest for this Journal and could contribute to scholarly conversations around sexual diversity, media, pop culture and more.

 My reading of the essay suggests that the author(s) are concerned only about the LGBT representations of ads but that they did only with a particular framework, Spain. The comparison between countries could be interesting in future, even if there are different approaches for the same advertisers but identifying adaptation in same countries, etc. 

 Other suggestion or personal doubt, could the same topic be analysed in other kind of contents different to advertising, for example films, series or videoclips?

 I would be find a specific discussion in that section with the mirror theory. I am impressed with this theory linked with this topic so... it is a strength as theoretical contribution as a start point for further discussion.

Is the work clearly and accurately presented and does it cite the current literature?

Yes

If applicable, is the statistical analysis and its interpretation appropriate?

Not applicable

Are all the source data underlying the results available to ensure full reproducibility?

Yes

Is the study design appropriate and is the work technically sound?

Yes

Are the conclusions drawn adequately supported by the results?

Yes

Are sufficient details of methods and analysis provided to allow replication by others?

Yes

Reviewer Expertise:

Happiness management, organizational comunication

I confirm that I have read this submission and believe that I have an appropriate level of expertise to confirm that it is of an acceptable scientific standard.
